# Top-Level Categories of Constitutively Organized Material Entities - Suggestions for a Formal Top-Level Ontology

**DOI:** 10.1371/journal.pone.0018794

**Published:** 2011-04-21

**Authors:** Lars Vogt, Peter Grobe, Björn Quast, Thomas Bartolomaeus

**Affiliations:** Institut für Evolutionsbiologie und Ökologie, Universität Bonn, Bonn, Germany; University College Dublin, Ireland

## Abstract

**Background:**

Application oriented ontologies are important for reliably communicating and managing data in databases. Unfortunately, they often differ in the definitions they use and thus do not live up to their potential. This problem can be reduced when using a standardized and ontologically consistent template for the top-level categories from a top-level formal foundational ontology. This would support ontological consistency within application oriented ontologies and compatibility between them. The Basic Formal Ontology (BFO) is such a foundational ontology for the biomedical domain that has been developed following the single inheritance policy. It provides the top-level template within the Open Biological and Biomedical Ontologies Foundry. If it wants to live up to its expected role, its three top-level categories of material entity (i.e., ‘object’, ‘*fiat* object part’, ‘object aggregate’) must be exhaustive, i.e. every concrete material entity must instantiate exactly one of them.

**Methodology/Principal Findings:**

By systematically evaluating all possible basic configurations of material building blocks we show that BFO's top-level categories of material entity are not exhaustive. We provide examples from biology and everyday life that demonstrate the necessity for two additional categories: ‘*fiat* object part aggregate’ and ‘object with *fiat* object part aggregate’. By distinguishing topological coherence, topological adherence, and metric proximity we furthermore provide a differentiation of clusters and groups as two distinct subcategories for each of the three categories of material entity aggregates, resulting in six additional subcategories of material entity.

**Conclusions/Significance:**

We suggest extending BFO to incorporate two additional categories of material entity as well as two subcategories for each of the three categories of material entity aggregates. With these additions, BFO would exhaustively cover all top-level types of material entity that application oriented ontologies may use as templates. Our result, however, depends on the premise that all material entities are organized according to a constitutive granularity.

## Introduction

Biomedical databases are becoming increasingly important and more and more researchers and health professionals use them on a daily basis for storing, annotating, managing, sharing, and analyzing their data and metadata. The highest possible degree of interoperability and re-usability of the contents of databases requires the development of commonly accepted standards for data and metadata – a process that already has been initiated in various biomedical communities (e.g. [1]). Ontologies thereby play an important role (e.g. [2]–[4]), as they have the potential to provide controlled vocabularies with explicit definitions (i.e. concept standards) and unambiguous designations (i.e. nomenclatural standards). In addition with a standardized format that is highly formalized and thus computer-parsable (i.e. format standard), they provide three of four very important components of any data and metadata standard ([5]). Biomedical ontologies are thus becoming increasingly important and are believed to be useful not only in the standardization of data and metadata, but also for data integration, data compatibility and comparability, and for the communication and management of data (for an overview of currently available biomedical ontologies see BioPortal, http://bioportal.bioontology.org).

Unfortunately, however, many biomedical ontologies fail to live up to these claims, since their definitions are not comparable and/or compatible among each other. This is partly due to the fact that most ontologies are application oriented and have been developed with a particular practical purpose in mind. As a consequence, a lot of attention went into the development of definitions for very specialized types of entities, whereas for general types explicit definitions are often lacking. Frequently, this causes ontological inconsistencies within the ontology and incompatibilities between different ontologies. In order to circumvent these problems, top-level formal foundational ontologies have been proposed that provide a standardized and ontologically consistent framework for the top-level categories of application oriented ontologies (e.g. [6]–[9]).

The Basic Formal Ontology (BFO, http://www.ifomis.org/bfo; [10]) represents a very general formal top-level ontology that has been developed as a realist ontology (i.e. representing kinds of entities and their divisions that exist in the mind-independent world) with the primary intention to be used in the structuring of scientific biomedical domain ontologies [11], as for example within the framework of the Open Biological and Biomedical Ontologies Foundry (OBO Foundry, http://www.obofoundry.org), which is one of the most important ontology repositories of the biomedical domain. BFO is intended to be used as the top-level template for all the biomedical ontologies listed in the OBO Foundry. As a top-level ontology, BFO does not contain physical, chemical, biological or other terms, which would properly fall within the special sciences domains. An increasing number of ontologies are becoming available that use BFO for their top-level framework (http://www.ifomis.org/bfo/users).

BFO has been developed in accordance with the *single inheritance policy*: all its defined categories are disjoint and exhaustive; they aim at being mutually exclusive relative to a given level of granularity [11]. In other words, each class has maximally one single asserted parent class. Thus, whereas a material object at one level of granularity may be an aggregate of objects at a finer level of granularity, it cannot be both an object and an aggregate of objects at the same level of granularity. The single inheritance policy thereby supports clear statements of definitions, easier and more reliable ontology curation, and it allows using more powerful reasoning tools and a single measure of distance between two classes (e.g. [12]). Multiple inheritance, in contrast, often goes hand in hand with errors in ontology construction (e.g. [13]) and can substantially complicate and even prohibit coherent integration across ontologies (e.g. [14]).

**Figure 1:**
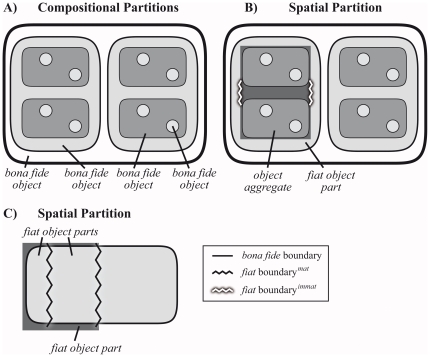
Compositional and spatial partitions. **A**. A *compositional partition* of *bona fide* objects that are situated in a nested fashion (constitutional hierarchy; see Fig. 2) within *bona fide* objects of a coarser granularity, which in their turn are, again, situated within *bona fide* objects of an even coarser granularity. A compositional partition always yields objects that are demarcated exclusively by *bona fide* boundaries. **B**. A *spatial partition* of the same constitutively organized *bona fide* object into an object aggregate and a *fiat* object part. **C**) A *spatial partition* of a *bona fide* object resulting in two *fiat* object parts, one of which in its turn is spatially partitioned again, resulting in two other *fiat* object parts. ***Fiat*** **boundary** ***^mat^*** **:** demarcates *fiat* parts of a material entity; ***fiat*** **boundary** ***^immat^*** **:** demarcates *fiat* parts of an immaterial entity (i.e. a hole).

If BFO wants to live up to its role as the provider of a formal top-level ontology for scientific biomedical domain ontologies, then its top-level categories must be mutually exhaustive and disjoint within a given level of granularity. Therefore, for any given level of granularity, a material entity must instantiate exactly one of the three types of material entity that BFO defines: *fiat* object part, object, or object aggregate (for definitions see table 1). A look at real material entities from the biomedical domain, however, reveals a lack of exhaustiveness. Gupta et al. [15] for instance, noted that in their ontological database for subcellular neuroanatomy they had to allow for multiple inheritance of BFO categories of material entity, because otherwise they could not have consistently classified all relevant biological entities. For example, they defined ‘synapse’ as a cell junction (i.e. a portion of extracellular space that thus has no demarcated boundaries) *“where axon terminals and dendritic processes are situated (hence it is an* [object] *aggregate) closely enough such that chemical neurotransmitters can pass from the axon terminals to the neurotransmitter receptor portions (e.g., post-synaptic density) of those dendrites”* ([15], p. 69; see also Figure 1 therein). Thus, according to Gupta et al. [15], on a cellular level of granularity, synapses are both object aggregates and *fiat* object parts. They noted that ‘synapse’ was not the only example and that they had to allow for such multiple inheritance also for other neuroanatomical entities, as for instance gap junctions or the node of Ranvier. Obviously, BFO's top-level categories of material entity are not exhaustive nor mutually disjoint.

**Table 1:** Definitions of the basic types of material entity of the Basic Formal Ontology (BFO version 1.1).

Definition	Parent Class Affiliation	Link/ID
**‘material entity’:** *“An independent continuant that is spatially extended whose identity is independent of that of other entities and can be maintained through time.* *Note: Material entity subsumes object, fiat object part, and object aggregate, which assume a three level theory of granularity, which is inadequate for some domains, such as biology.* *Examples: collection of random bacteria, a chair, dorsal surface of the body”*	‘independent continuant’	http://www.ifomis.org/bfo/1.1/snap#MaterialEntity
**‘object’:** *“A material entity that is spatially extended, maximally self-connected and self-contained (the parts of a substance are not separated from each other by spatial gaps) and possesses an internal unity. The identity of substantial object entities is independent of that of other entities and can be maintained through time.* *Examples: an organism, a heart, a chair, a lung, an apple”*	‘material entity’	http://www.ifomis.org/bfo/1.1/snap#Object
**‘fiat object part’:** *“A material entity that is part of an object but is not demarcated by any physical discontinuities.* *Examples: upper and lower lobes of the left lung, the dorsal and ventral surfaces of the body, the east side of Saarbruecken, the lower right portion of a human torso”*	‘material entity’	http://www.ifomis.org/bfo/1.1/snap#FiatObjectPart
**‘object aggregate’:** *“A material entity that is a mereological sum of separate object entities and possesses non-connected boundaries.* *Examples: a heap of stones, a group of commuters on the subway, a collection of random bacteria, a flock of geese, the patients in a hospital”*	‘material entity’	http://www.ifomis.org/bfo/1.1/snap#ObjectAggregate

In the following we systematically evaluate and assess the exhaustiveness of BFO's top-level categories of material entity. By referring to adequate examples from biology and everyday life we demonstrate the necessity of two additional top-level categories, which we introduce and discuss. We also suggest additional subcategories, which we believe provide valuable top-level classes for biomedical domain ontologies. We conclude by making suggestions for extending BFO to meet the single inheritance principle.

## Results

### Boundaries and Entities

#### Two Types of Boundaries: Bona Fide and Fiat

The moon, an apple, you and I – not only are all these entities extended in space, but they all can be clearly and unambiguously demarcated from their respective environments and complements (i.e. the universe without the moon, without this apple, without you, or without me). Each of these entities possesses a single continuous outer boundary that we usually recognize as its outer two-dimensional surface. This surface, a boundary that clearly belongs to the entity, not only demarcates it from its complement, but also the complement from the object. It is therefore a symmetrical demarcation [16]. Since the boundary is only possessed by the entity and not its complement, the entity is closed and its complement is open [16]. Therefore, boundaries demarcating material from immaterial entities (i.e. negative objects, holes), for instance those demarcating cups from their holes, are only possessed by the material hosts but not by the immaterial entities themselves [16], [17].

Outer boundaries of material entities can be demarcated on grounds of spatial discontinuity or qualitative heterogeneity (e.g., material constitution, color, texture, or electric charge) and are commonly called *bona fide* boundaries [16], [18], [19]. All *bona fide* boundaries are characterized by qualitative differentiations or discontinuities and thus are physical boundaries that exist independently of all human cognitive acts [18]–[20]. River-banks, coastlines, the surface of a cell membrane, the surface of my entire body or of a football are all examples of *bona fide* boundaries.

The moon, an apple, you and I – each of these entities is not only an object that exists extended in space. It also consists of divisible matter that can be divided along inner boundaries into spatial parts – in reality and in thought. Just like outer boundaries, *bona fide* inner boundaries presuppose an interior spatial discontinuity or a qualitative heterogeneity among its parts [16], [18]. In humans, for instance, two-dimensional inner boundaries demarcate particular organs, cells, or molecules from one another, whereas one-dimensional inner boundaries demarcate specific regions of a surface along for instance edge-lines of an eyelid or a lip. However, organisms can be divided also along inner boundaries that are not *bona fide* boundaries. Such boundaries are commonly called *fiat* boundaries, because they are non-physical boundaries that exclusively depend on acts of human decision – they are the product of our mental and linguistic activity and represent only potential boundaries (i.e. they do not actually separate anything in reality), owing their existence to associated conventional laws, political decrees or habits, or to related human cognitive phenomena [16], [19]–[21]. Examples of *fiat* boundaries include the Equator, the North Pole, the boundaries of postal districts, the inner boundary demarcating my head from the rest of my body, or the *fiat* boundary of a mountain that demarcates the mountain from the ground underneath it.

Although arbitrary, *fiat* boundaries nevertheless may be determined by specific (*bona fide*) landmarks or coordinates, which are required for reliably re-locating *fiat* boundaries; [19], [20], [22], or other pragmatic or even scientifically justified reasons. Contrary to *bona fide* boundaries, which are always owned by their respective *bona fide* objects and not their complements, *fiat* boundaries are shared by all *fiat* parts involved: the Equator belongs to both the northern and the southern hemisphere, or each hemisphere has its own Equator and the two Equators *coincide* [16], [21]. Only *fiat* boundaries coincide.

Thereby it seems reasonable to distinguish two types of *fiat* boundary (see also [23]): (i) *fiat* boundaries*^mat^* that demarcate *fiat* parts of material entities and (ii) *fiat* boundaries*^immat^* that demarcate *fiat* spaces (i.e. immaterial entities, negative objects), like for instance tunnels, which are not bounded on all sides in *bona fide* fashion by their supporting material hosts, but also possess an entrance and an exit that are demarcated by *fiat* boundaries*^immat^* [20]. The same applies to caves and hollows that always possess an entrance that is demarcated by a *fiat* boundary*^immat^* (the only type of hole that is not demarcated by a portion of *fiat* boundary*^immat^* are closed cavities).

Despite their fundamental ontological differences, both *fiat* and *bona fide* boundaries cannot exist independently of the entities that they bound – they ontologically depend on their higher-dimensional hosts [16], [19]. The categorical distinction between *bona fide* and *fiat* boundaries, however, is considered to be absolute – while *fiat* boundaries mark potential *bona fide* boundaries of an object, they never turn into *bona fide* boundaries themselves, but can only be considered to *precede* them in time in case their *bona fide* counterparts emerge as a result of some ‘cutting/dividing’ event in the future [16].

#### Two Types of Material Entity: Object and Fiat Object Part

On the basis of the distinction of *fiat* and *bona fide* boundaries one can distinguish two types of material entity: (i) *bona fide object*, which possesses a *single* continuous *bona fide* outer boundary, and (ii) *fiat object part*, which possesses *some fiat* outer boundary*^mat^* [16], [18]–[21]. Whereas the existence of *bona fide* objects is independent of human cognitive activities, the recognition and establishment of *fiat* inner boundaries*^mat^* is of crucial importance for the recognition of *fiat* object parts – their existence depends on human cognitive acts. A *fiat* inner boundary*^mat^* of a *bona fide* object is the *fiat* outer boundary*^mat^* of one of the object's *fiat* object parts. Examples for *fiat* object parts are the northern hemisphere, my left foot, a mountain, or the branch of a tree.

Moreover, since *bona fide* objects possess a single continuous *bona fide* outer boundary and are thus closed entities, and since contact (in terms of connection or coincidence) between two closed entities is, at least from a strictly topological point of view, not possible [16], [20], [21], aggregates of *bona fide* objects cannot be intrinsically connected and thus would have to form (more or less far) scattered wholes [16], [18]–[20] (we introduce a more differentiated view further below).

Although *fiat* boundaries are created by us and, as a consequence, the demarcation of *fiat* entities depends on human fiat, *fiat* entities themselves are nevertheless autonomous portions of reality and are ‘objective’ in this sense [18].

### Partitioning, Basic Formal Ontology, and Constitutive Granularity

Entities that are extended in space can be partitioned along the lines of *bona fide* and *fiat* inner boundaries. As a consequence, one can distinguish two types of partitions [23]–[26]. (i) Spatial (or *fiat*) partitions that partition a given entity into regional *fiat* parts (i.e. *fiat* object parts or object aggregates) along the lines of *fiat* inner boundaries*^mat^* ^,*immat*^ (and possibly some *bona fide* boundaries), as for instance the partition of a human body into head, trunk, and extremities. The regional parts that result from a spatial partitioning originate from an arbitrary subdivision of an object into constitutional *fiat* parts that share a given location within and relative to the object. On the other hand, (ii) compositional partitions that partition a given entity into its constitutional parts exclusively along the lines of *bona fide* boundaries, as for instance a human body into its various organs (Fig. 1).

The parts that result from both spatial and compositional partitioning can be partitioned again: *fiat* object parts can be spatially partitioned along *fiat* boundaries*^mat^* into ever smaller *fiat* parts (and this can be done innumerable times – at least in theory) or they can be compositionally partitioned into their constitutional *bona fide* objects along *bona fide* boundaries. The same applies to *bona fide* objects and object aggregates, too. Therefore one can always distinguish three levels of granularity for any type of *bona fide* object (in accordance with BFO, see notes to ‘material entity’ in table 1): (i) the granularity level of the *bona fide* object itself, (ii) a finer granularity level of its *fiat* object parts, and (iii) a coarser level of aggregates of *bona fide* objects:

*fiat object part<object<object aggregate.*

**Figure 2:**
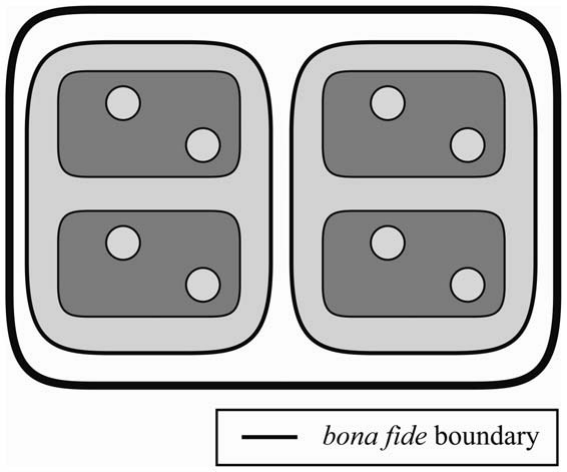
Constitutive granularity. A *constitutive granularity* of, for example, molecules, cells and organs of a multicellular organism. In constitutive granularities, all objects belonging to one level of granularity are parts of objects of the next higher level of granularity: all molecules are part of cells, all cells part of organs, and all organs part of multicellular organisms. Moreover, summing all objects of one level together yields the maximal object – here, a multicellular organism.

This very simple granularity scheme becomes more complicated when we allow different types of objects to belong to different granularity levels and objects of finer granularity to be parts of objects of coarser granularity. In case object aggregates constitute *bona fide* objects of coarser granularity (e.g. an aggregate of atoms constituting a molecule), one receives a constitutive hierarchical organization (see Fig. 2) of *bona fide* objects of different granularity that are nested within one another – a constitutive granularity (see also constitutive hierarchy [27], [28]). Most granularity schemes suggested so far presuppose that all types of material entity are constitutively organized (e.g. [24], [29]; for an exception see [26]), with the consequence that:

- higher level entities consist of physically joined elements,
- all objects belonging to one level of granularity form parts of objects of the next higher level of granularity,
- summing all objects together that belong to one level of granularity yields a maximal *bona fide* object – in other words, all the parts that share the same granularity level exhaustively sum to the whole (e.g. summing together all cells of a human individual yields the entire human body).

**Figure 3:**
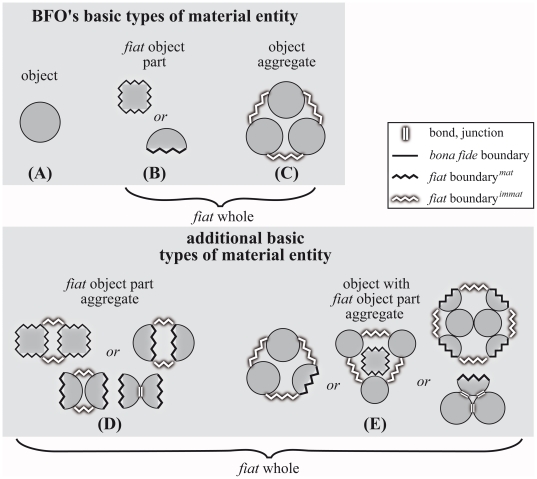
First order basic types of material entity. **A–C**. The three different basic types of material entity that the Basic Formal Ontology (BFO) currently distinguishes. If the distinction between *fiat* and *bona fide* boundaries is taken to be absolute across all levels of granularity, object aggregates are always demarcated by *fiat* boundaries*^immat^* and thus always represent *fiat* wholes – but see the distinction between metric proximity, adherence and coherence and the distinction between clusters and groups in the text. **D** & **E**. Two additional basic types of material entity that are currently not recognized by BFO. With the exception of ‘object’, all types possess some *fiat* boundary and thus are *fiat* wholes. ***Fiat*** **boundary** ***^mat^*** **:** demarcates *fiat* parts of a material entity; ***fiat*** **boundary** ***^immat^*** **:** demarcates *fiat* parts of an immaterial entity (i.e. a hole).

Assuming a constitutive organization of material entities brings about some consequences:

1. One could, for instance, partition a human body into the following granularity levels, ordered from finer to coarser grained levels:
  [*fiat atom part*<*atom*<*atom aggregate*]<[*fiat molecule part*<*molecule*<*molecule aggregate*]<[*fiat cell part*<*cell*<*cell aggregate*]<[*fiat organ part*<*organ*<*organ aggregate*]<[*fiat body part*<*body*<*body aggregate*].
  Thereby, the general granularity scheme remains three-leveled in the sense that every distinguishable *bona fide* ‘object’ level has its corresponding ‘*fiat* object part’ level and ‘object aggregate’ level associated. The granularity relations between corresponding *fiat* object parts, objects, and object aggregates (i.e. inside a pair of brackets, e.g. between a particular *fiat* cell part and a cell or a particular cell and a cell aggregate), as well as those between different types of objects (i.e. across brackets, e.g. between a particular atom and a particular molecule), can be determined universally. The other granularity relations (i.e. across brackets, between different top-level types of material entity; e.g. between a particular cell aggregate and a particular *fiat* organ part), however, cannot. This follows directly from the constitutive granularity of material entities: an atom, for instance, can be (at a finer level) an object in its own right and (at a coarser level) a *fiat* object part of a molecule; and a molecule simultaneously an object, a *fiat* object part of a cell and a *fiat* object part of an organ. Therefore, regarding granularity, these relations (i.e. relations across brackets) have to be decided on a case by case basis (see [26]).
2. In constitutively organized material entities, molecules are composed of atoms, cells of molecules, and organisms of cells. According to the above mentioned theory of boundaries, however, objects cannot be topologically connected to one another, because, according to Smith & Varzi [16], [21] (see also [20]), there is always a small gap between two objects – at least from a strictly topological point of view. Several objects together can only form object aggregates, and object aggregates are demarcated by non-connected boundaries (see definition provided by BFO; table 1). Therefore, object aggregates would always have to include some *fiat* boundary*^immat^* through the space that separates the object entities from one another (see Fig. 3C). Consequently aggregates of *bona fide* objects of a finer level of granularity could not constitute *bona fide* objects at coarser levels, because *bona fide* objects require that their parts are not separated by gaps. This is a problem that arises from the specific ontological notion of self-connectedness that BFO's definition of ‘object’ refers to (a problem that has been noticed before; e.g. [30]). This would prohibit, for instance, cells and multicellular organisms to be *bona fide* objects, because they are composed of molecules. Yet, multicellular organisms and cells are generally considered to represent prototypical *bona fide* objects. If we want them to keep this status, the distinction between object, *fiat* object part, and object aggregate cannot be absolute across all levels of granularity, and neither can the distinction between *bona fide* and *fiat* boundaries. In other words, in order to do justice to a constitutive organization of material *bona fide* objects in reality, the notion of *fiat* and *bona fide* boundary must be granularity dependent: what is a *bona fide* boundary at a finer level of granularity may be *fiat* at a coarser. This, however, would imply that we have to distinguish two different types of object aggregate as well (see *group* and *cluster* discussed later).

### A Scheme of Top-Level Categories of Constitutively Organized Material Entities

Considering the foundational role that BFO claims to take for the scientific domain, an important question is whether its distinction of three basic types of material entity (i.e. *fiat* object part, object, object aggregate) is (i) exhaustive and (ii) sufficiently differentiated and specific. In other words, (i) *is there evidence for material entities that cannot be subsumed under one of the three suggested basic types*, and (ii) *is there evidence documenting the need for differentiation of further subtypes of the suggested basic types?*

#### Exhaustiveness

**Figure 4:**
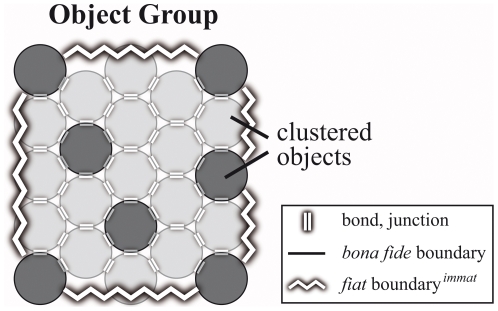
Object groups are spatially scattered *fiat* entities. An object group is an aggregate of objects in which the objects (here shown in dark grey) are separated from each other by space or, like in the case depicted, by other objects (shown in light grey), with which they can even form an object cluster. Every object group is demarcated by a combination of *bona fide* boundaries and *fiat* boundaries*^immat^*. The example depicted could represent the distribution pattern of sensory cells (i.e. sensory cell group) within an epithelial cell cluster, in which the sensory cells (dark grey) are part of the sensory cell group as well as of the epithelial cell cluster. ***Fiat*** **boundary** ***^immat^*** **:** demarcates *fiat* parts of an immaterial entity (i.e. a hole).

*Is there evidence for material entities that cannot be subsumed under one of the three suggested basic types?* This question can be answered by considering the fundamental ontological assumption that underlies the basic categorization of material entities in BFO: the existence and distinction of two fundamentally different types of boundaries – *bona fide* and *fiat* boundaries. From this distinction follows the differentiation of two spatio-structural **building blocks** for all kinds of material entity, (i) *bona fide* objects and (ii) *fiat* object parts. They represent building blocks, because every material entity is either a *bona fide* object, a *fiat* object part, or a combination thereof. Since the distinction between *fiat* and *bona fide* boundaries is absolute and exhaustive [16], [19], so is the inventory of spatio-structural building blocks. However, the inventory of building blocks does not equal the inventory of different types of material entity which can exist – like an inventory of different types of Lego bricks, the inventory of building blocks only lists all those types of basic entities, of which all kinds of material entity are built. And just as various different types of structures can be built from Lego bricks, various different types of material entity can be built from different combinations of *bona fide* objects and *fiat* object parts – at least in theory. In order to receive an exhaustive list of theoretically possible basic types of constitutively organized material entities one thus only has to permute all possible combinations of *bona fide* objects and *fiat* object parts and their distribution in space. This results in the schemes of possible types of basic material entity shown in Figures 3 and 4. They cover all theoretically possible types of combinations of building blocks and their possible types of distribution in space. Given that all material entities are constitutively organized, the list of types presented in these schemes is, thus, exhaustive. In the following we present the additional types of material entity and discuss their necessity as top-level categories.

#### Additional Top-Level Category: ‘Fiat Object Part Aggregate’

One possible combination of building blocks is two or more *fiat* object parts constituting a ***fiat*** **object part aggregate** (Fig. 3D, table 2).

**Table 2:** Definitions of additional basic types of material entity.

Definition	Parent Class Affiliation
**‘fiat object part aggregate’:** *A material entity that is a mereological sum of separate (i.e. not sharing a fiat boundary with each other) fiat object part entities and possesses non-connected fiat boundaries.* *Examples: a synapse, the fingers of a hand, a joint, a door hinge, hydrogen bond between molecules, an estuary, mainland of the Russian Federation, mainland of Turkey*	‘material entity’
**‘object with fiat object part aggregate’:** *A material entity that is a mereological sum of separate (i.e. not sharing a fiat boundary with each other) object and fiat object part entities and possesses non-connected boundaries.* *Examples: a human heart, a power outlet, a train station, a traditional telephone cord connection between two telephones, the territories of Turkey and of England*	‘material entity’
**‘object group’:** *An object aggregate that is a mereological sum of spatially separated object entities, which do not adhere to one another through chemical bonds or physical junctions but, instead, relate to one another merely on grounds of metric proximity. The objects can be separated from one another through space or through other object entities that do not belong to the group* *Examples: a heap of stones, a colony of honeybees, the trees of a forest, the fish of a shoal, a group of commuters on the subway, the patients in a hospital*	‘object aggregate’
**‘object cluster’:** *An object aggregate that is a mereological sum of separate object entities, which adhere to one another through chemical bonds or physical junctions that go beyond gravity.* *Examples: the atoms of a molecule, the molecules forming the membrane of a cell, the epidermis in a human body*	‘object aggregate’
**‘fiat object part group’:** *A fiat object part aggregate that is a mereological sum of spatially separated fiat object part entities, which do not adhere to one another through chemical bonds or physical junctions but, instead, relate to one another merely on grounds of metric proximity. The fiat object parts can be separated from one another through space or through other material entities that do not belong to the group* *Examples: the fingers of a hand, a joint, a door hinge, opposite riverside sections, mainland of the Russian Federation*	‘fiat object part aggregate’
**‘fiat object part cluster’:** *A fiat object part aggregate that is a mereological sum of separate fiat object part entities, which adhere to one another through chemical bonds or physical junctions that go beyond gravity.* *Examples: synapse, hydrogen bond between molecules, an estuary, mainland of Turkey*	‘fiat object part aggregate’
**‘object with fiat object part group’:** *An object with fiat object part aggregate that is a mereological sum of spatially separated object entities and fiat object part entities, which do not adhere to one another through chemical bonds or physical junctions but, instead, relate to one another merely on grounds of metric proximity. The objects and fiat object parts can be separated from one another through space or through other material entities that do not belong to the group* *Examples: the equilibrium organ of a lobster, a modern wireless cell phone connection, the territories of Turkey and of England*	‘object with fiat object part aggregate’
**‘object with fiat object part cluster’:** *An object with fiat object part aggregate that is a mereological sum of separate object entities and fiat object part entities, all of which adhere to one another through chemical bonds or physical junctions that go beyond gravity.* *Examples: a human heart, a power outlet, a train station, a traditional telephone cord connection between two telephones, a polyplacophoran aesthete*	‘object with fiat object part aggregate’

**Definition:** *A* ***fiat object part aggregate*** *is a material entity that is a mereological sum of separate fiat object part entities and possesses non-connected boundaries.*

**Explanation:** *Fiat* object part aggregates are demarcated by a combination of different types of boundaries (Fig. 3D). Since every *fiat* object part entity necessarily possesses some *fiat* boundary*^mat^*, all aggregates of *fiat* object parts will necessarily possess *fiat* boundaries*^mat^* as well. Many *fiat* object part entities, however, also possess portions of *bona fide* boundaries (Fig. 3B). Moreover, the *fiat* object part entities of the aggregate can be separated by gaps, in which case they are topologically positioned separate from, and relative to one another within space. This possible constellation holds for any aggregate of material entities: every aggregate of material entities can possess immaterial parts (i.e. negative objects: certain types of holes, e.g., tunnels, caves, tubes and hollows), which are continuously connected to the space surrounding the aggregate. Therefore, aggregates of material entities, and thus also *fiat* object part aggregates, can be demarcated by some *fiat* boundary*^immat^* (Fig. 3C–E). As a consequence, depending on the types of *fiat* object part entities that constitute the *fiat* object part aggregate and their position and orientation within the aggregate, a *fiat* object part aggregate may be demarcated by portions of *bona fide* boundary and *fiat* boundary*^immat^*, but is necessarily always demarcated by some *fiat* boundary*^mat^* (Fig. 3D).

**Examples:** In biology, a synapse is commonly considered to be an intercellular junction that is composed of the presynaptic zone of a neuron (i.e. a *fiat* cell part) and the postsynaptic zone of another neuron, muscle cell or secretory cell (i.e. another *fiat* cell part) with an intervening synaptic cleft (i.e. intercellular space) between the two zones (see *Introduction*). Synapses are thus *fiat* object part aggregates (see also [15]). There are several other examples of *fiat* object part aggregates from biology, as for instance the fingers of my left hand, a joint or articulation, or ciliary bands used for locomotion in various planktonic organisms.

In physics and chemistry the binding between positively and negatively charged electric poles of molecules or the chemical bindings between atoms within a molecule are examples of *fiat* object part aggregates in the physical domain. When we talk about an estuary, we usually refer to those parts of a river and sea which continuously merge into one another along with its accompanying riverbank and coastline areas. Thus, an estuary is an example of a *fiat* object part aggregate in the geographical domain. The continental landmasses of the Russian Federation with its exclave Kaliningrad Oblast is another example of a *fiat* object part aggregate in the geographical domain. In everyday life we also talk about *fiat* object part aggregates – for instance, if we talk about the four legs of a particular chair that is made out of one continuous and homogeneous piece of plastic, or when talking about a door hinge.

It is particularly noticeable that all the *fiat* object part aggregates in the examples are functional/causal elements that play an important role within some specific causal framework. The scientific domain often concerns itself with causal properties of fiat object part aggregates, which is why we require terms to be able to talk about them.

*Can ‘fiat object part aggregate’ be subsumed under one of the BFO types?* Obviously, *fiat* object part aggregates are not (*bona fide*) objects. The examples given above are not covered by BFO's ‘*fiat* object part’ or ‘object aggregate’. The synapse example discussed in the introduction [15] already suggests that *fiat* object part aggregates can neither be unambiguously subsumed under ‘object aggregate’ nor under ‘*fiat* object part’. An aggregate of *fiat* object parts possesses properties of both categories: it consists of parts that are parts of objects, it is a mereological sum of separate material entities, it is demarcated by some *fiat* boundary, and it possesses non-connected boundaries. However, while possessing non-connected boundaries, an aggregate of *fiat* object parts is not a mereological sum of separate object entities, but instead of *fiat* object parts and thus cannot be subsumed under BFO's category ‘object aggregate’. Neither is an aggregate of *fiat* object parts *necessarily* part of one particular object entity and it can possess physical discontinuities – it can be an aggregate of *fiat* object parts of several spatially separated object entities (Fig. 3D).

#### Additional Top-Level Category: ‘Object with Fiat Object Part Aggregate’

Besides the types of aggregates that are uniformly composed out of one type of building block, there is also the possibility of a type of aggregate that is composed of both types of building blocks, the **object with** ***fiat*** **object part aggregate**.

**Definition:** *An* ***object with fiat object part aggregate*** *is a material entity that is a mereological sum of separate object and fiat object part entities and possesses non-connected boundaries.*

**Explanation:** Object with *fiat* object part aggregates are demarcated by a combination of different types of boundaries (Fig. 3E). Since an object with *fiat* object part aggregate consists of both object and *fiat* object part entities, it will necessarily be demarcated by their typical types of boundaries, i.e. *fiat^mat^* and *bona fide* boundaries. Moreover, as already discussed above, the component entities of any aggregate of material entities can be spatially separated. Therefore, object with *fiat* object part aggregates can also possess some *fiat* boundary*^immat^* (Fig. 3E).

**Examples:** Most human organs (e.g. heart) are object with *fiat* object part aggregates, as they usually include various vessels (i.e. blood vessels, lymphatic vessels) through which they are connected to other organs within the human body. These connections allow the exchange of essential substances between different organs: supplies of nutrients, energy, and oxygen, as well as the disposal of metabolic waste products. Furthermore, they do contain a meshwork of nerve fibers, which are connected to the entire nervous system for the communication between the various functional elements within the human body. These fibers and vessels are *fiat* parts within the otherwise *bona fide* demarcated organ and together form an object with *fiat* object part aggregate. The nervous tissue of the spinal cord, which consists of complete neurons within the spine and *fiat* parts of the radiating spinal nerves is another example of an object with *fiat* object part aggregate. There are also examples of object with *fiat* object part aggregates from the geographical domain. The territories of Turkey and England, for example, consist of a mainland area, which is a *fiat* object part of the landmass of the respective continent (in case of Turkey, the mainland area itself is an aggregate of *fiat* object parts of the Asian and European landmass, separated by the Bosphorus), and some bona fide islands.

Also from everyday life there are examples of aggregates of objects and *fiat* object parts: for instance (i) a power outlet or ceiling lamp, which is assembled out of a set of *bona fide* objects and connected to the general power supply through a *fiat* part of a wire, (ii) a train station with the part of the railroad network that runs through it, or (iii) a traditional telephone connection with two telephones connected through a part of the telephone cable network.

*Can ‘object with fiat object part aggregate’ be subsumed under one of the BFO types?* Aggregates of objects and *fiat* object parts themselves are not (*bona fide*) objects, and the examples given above are not covered by BFO's ‘*fiat* object part’ or ‘object aggregate’. An aggregate of objects and *fiat* object parts possesses properties of both categories: it consists of parts that are parts of objects, it is a mereological sum of separate material entities, it is demarcated by some *fiat* boundary, and it possesses non-connected boundaries. However, despite possessing non-connected boundaries (a characteristic of object aggregates, see table 1), an aggregate of objects and *fiat* object parts is not a mereological sum exclusively composed of separate object entities. Instead, it is composed of both objects *and fiat* object parts and thus cannot be subsumed under BFO's category ‘object aggregate’. Neither is an aggregate of objects and *fiat* object parts part of one particular object entity. It possesses physical discontinuities; it is an aggregate of several separated material entities (Fig. 3E).

#### Specificity and Degree of Differentiation: the Need for discriminating Groups and Clusters

Assuming a constitutive granularity of material entities, with *bona fide* objects of coarser granularity being composed out of *bona fide* objects of finer granularity (e.g. cells out of molecules) (see *Partitioning*, *Basic Formal Ontology*, *and Constitutive Granularity*), rises another problem. What is the difference between a heap of stones and an aggregate of pieces of an assembled table (i.e. table-legs screwed to a table top), cells of a multicellular organism, or an aggregate of atoms of a molecule? While all four of them are object aggregates, the topological relation between the stones is qualitatively different from the relation between the assembled pieces, the relation between the cells, and the relation between the atoms. A heap of stones is an object aggregate merely due to the metric (i.e. measurable and, thus, quantifiable) proximity of its stones to one another – no bonds exist between the individual stones and no coherence-forces other than gravity are in effect. Gravitation itself is a kind of bonding-force that is always in effect between material entities, and thus cannot be used as a criterion for distinguishing different types of material aggregates. In contrast, the atoms of a molecule not only form an object aggregate, in which the individual atoms (i.e. objects) can still be distinguished from one another through the spatial distribution of their nuclei, but due to chemical bonds between the atoms, they at the same time constitute a molecule and thus a *bona fide* object at a coarser granularity. The same holds for an assembled table, which forms an object aggregate at a finer grained level, but due to physical junctions (e.g. screws, nails, clinches, riveting bolts, welds, etc.), which hold the construction together, at the same time it also constitutes a *bona fide* piece of furniture at a coarser granularity. Similarly, the cells of a multicellular organism form an object aggregate, but due to the cell-cell junctions between them they also constitute a multicellular organism and thus a *bona fide* object at a coarser granularity.

The chemical bonds that adhere atoms of a molecule together and the physical junctions that adhere mesoscopic and macroscopic pieces together provide a degree of cohesion that goes beyond gravitation. The atoms of a molecule as well as the cells of a multicellular organism and the pieces of an assembled object form object aggregates *not merely* due to metric proximity, but much rather due to physical/chemical adherence. The degree of cohesion between the atoms of a molecule is weaker than between the atomic parts belonging to each of its atoms; likewise the degree of cohesion between cells of a cellular organism is weaker than between the molecules belonging to each of its cells.

Therefore, it is reasonable to distinguish *groups* and *clusters* of material entities. Groups of material entities are scattered material entity aggregates whereas clusters are lumped material entity aggregates. We need both categories in our research practice as well as in everyday life. For example, whenever we want to refer to an aggregate of material entities that exhibits a specific spatial distribution pattern of scattered material entities we are referring to a group. If we want to refer to an aggregate of material entities that forms a cohesive/connected whole consisting of several material entities we are referring to a cluster.

**Definition:** *A* ***group*** *is an aggregate of material entities that is a mereological sum of spatially separated material entities, which do not adhere to one another through chemical bonds or physical junctions but, instead, relate to one another merely on grounds of metric proximity.*

**Definition:** *A* ***cluster*** *is an aggregate of material entities that is a mereological sum of separate material entities, which adhere to one another through chemical bonds or physical junctions that go beyond gravity.*

**Explanation and Example for ‘group’:** Metric proximity implies the actual existence of gaps between the entities within the aggregate, and thus space that can even be occupied by objects that do not belong to the aggregate, resulting in spatially scattered entities (Fig. 4). The material entities of a group (see also *collection*, [31]; related to, but not identical with Smith's use of ‘group’ in [32] or ‘agglomeration’ in [33]) are positioned topologically separately from and relative to one another within space, like for instance trees in a forest, fish in a shoal, or a heap of stones. Thus, a group of material entities always encloses some part of space as well. In other words, every group of material entities possesses immaterial parts, which are continuously connected to the space surrounding the aggregate. As a consequence, every group of material entities is demarcated from its complement by some *fiat* boundary^immat^. After all, one of the characteristics of a forest is that it is composed of a group of trees with the trees being separated from each other by space. The resulting spatial arrangement of individual trees forms a characteristic pattern: without the spatial gaps between individual trees, no forest; without spatial gaps, no groups. These gaps may be occupied by other types of material entities or they may be “empty”, but this is irrelevant to their ontological nature as group. What is important, however, is the existence of gaps between the material entities (i.e. trees) that are *relevant* for the coarser entity of interest (i.e. forest).

**Explanation and Example for ‘cluster’:** Chemical bonds or physical junctions cause the entities of an aggregate to adhere to one another, as for instance the atoms of a molecule, the lipid molecules forming a cell membrane, or the cells forming an epidermis of a human body. Contrary to metric proximity, no spatial gap separates the material entities of a cluster (table 3). As a consequence, object clusters can build continuous boundaries for objects of coarser granularity levels, thereby marking the border of these higher level objects. The cell membranes of animal cells, for example, provide a clear demarcation of the cell towards its environment. The membrane itself is composed of a multiplicity of individual lipid-molecules, which, due to the hydrophobic properties of their C-tails and the hydrophilic properties of their heads, together form a bilayer. This bilayer is stabilized through van der Waals' forces between the C-tails. This lipid bilayer is a type of molecule cluster that provides the *bona fide* boundaries for all animal cells.

**Table 3:** Three foundational types of spatio-topological relations between material entities.

Type of material entity	Relation between its parts	Type of inner boundary separating its parts	Characteristics
object or *fiat* object part	topological coherence	*fiat* boundary^mat^ between *fiat* object parts	Coherence implies physical continuity and qualitative homogeneity within the object or *fiat* object part
object cluster	topological adherence	*bona fide* boundary between objects	Adherence implies physical continuity and qualitative heterogeneity within the object cluster
object group	metric proximity	*bona fide* boundary and *fiat* boundary^immat^ between objects	Metric proximity implies physical separation through spatial gaps between the constitutive objects of the object group

#### Distinguishing fiat and bona fide inner Boundaries: Coherence vs. Adherence

Since the material entities of a cluster are not topologically separated, they either topologically cohere or topologically adhere. Coherence implies topological connection between the material entities to which it applies. Two material entities that cohere to one another are topologically connected, and thus form a continuous coherent whole which can be demarcated only by *fiat* boundaries (table 3), like for instance an active center in an enzyme.

Adherence implies *topological* non-connection between material entities, but, contrary to metric proximity, requires some chemical or physical connection between the entities that adhere to each other. In other words, adherence implies topological contact between the material entities to which it applies. Whenever an inner *bona fide* boundary exists within a physically continuous object, this boundary is marked by a qualitative heterogeneity that results from adherence, as opposed to qualitative homogeneity that results from coherence. For instance the cells of a multicellular animal are demarcated by such inner *bona fide* boundaries: from a molecular point of view, the cell surfaces represent inner boundaries within a physically continuous object (i.e. multicellular animal) that are marked by qualitative heterogeneity (i.e. cell membranes).

**Figure 5:**
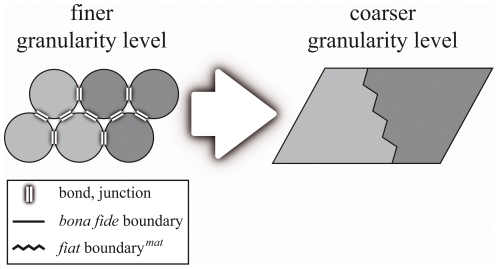
Granularity dependence of *bona fide* boundaries of some object clusters. **Left:** An object cluster consisting of six object entities. This cluster is exclusively demarcated by *bona fide* boundaries, and so is any of its sub-clusters (e.g. the two sub-clusters, each consisting of three object entities (in light-grey and in dark-grey). **Right:** The object cluster consisting of six object entities constitutes an object at a coarser granularity level. This object is demarcated from its surrounding complement by a *bona fide* boundary. Contrary to the finer granularity level, however, within this coarser level the two sub-clusters cannot be demarcated by *bona fide* boundaries anymore: The adherence relation between the objects involved (light-grey and dark-grey) at the finer level maps to a coherence relation at the coarser granularity level. Therefore, the respective parts are demarcated by a *fiat* boundary*^mat^*. ***Fiat*** **boundary** ***^mat^*** **:** demarcates *fiat* parts of a material entity.

On a higher level of granularity, the adherence through chemical bonds or physical junctions between material entities of finer granularity is treated as coherence (Fig. 5): a given material entity may be treated as an aggregate of two cells that adhere to one another at the finer cellular level of granularity, and at the same time as a *fiat* body part at the coarser multicellular-organism level. On the level of a multicellular organism, the cells form a continuous matter in which single cells are considered to cohere rather than adhere to one another and, at least on this level, any division of cell aggregates is treated as being a *fiat* body part.

Topological adherence and topological coherence always involve other cohesion forces than only gravitation. Since the distinction between gravitation and all other physicochemical cohesion forces (e.g. electromagnetic forces) is *bona fide* (i.e. mind independent), the distinction between topological adherence and topological coherence on the one hand and metric proximity on the other hand is *bona fide* as well. As a consequence, the differentiation of material entity aggregates into clusters and groups of material entities is *bona fide* and categorial.

#### Additional Top-Level Categories resulting from discriminating Groups and Clusters

**Figure 6:**
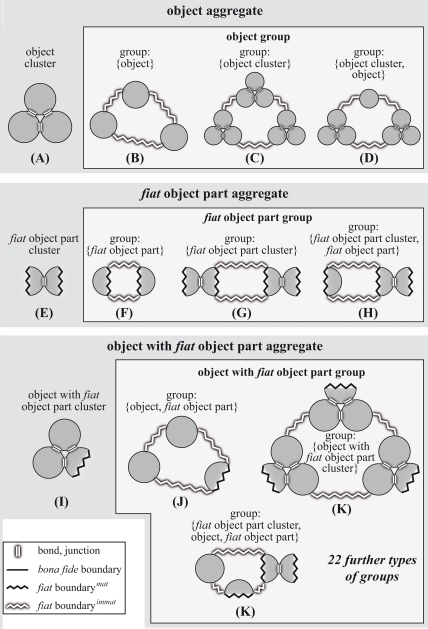
Second order basic types of material entity. Possible subcategories of the three basic types of aggregates that can be differentiated on grounds of distinguishing two types of relation between the objects within the aggregate (i.e. metric proximity and adherence) and the presence or lack of *fiat* boundaries*^immat^*: (i) clusters *are not* demarcated by *fiat* boundaries*^immat^* and are further characterized by topological adherence between the entities of the aggregate (through chemical bonds or physical junctions); (ii) groups *are* demarcated by *fiat* boundaries*^immat^* and are further characterized merely by metric proximity of the entities of the aggregate – they lack adherence. Since also clusters can spatially relate to one another on grounds of metric proximity, clusters can also be part of groups. **A–D**. The four basic types of object aggregate – one object cluster and three types of object group, all of which either consist of objects, object clusters, or both. Note that an object cluster is only demarcated by *bona fide* boundaries and thus does not represent a *fiat* whole. **E–H**. The four basic types of *fiat* object part aggregate – one *fiat* object part cluster and three types of *fiat* object part group, all of which either consist of *fiat* object parts, *fiat* object part clusters, or both. **I–K**. Four out of 26 basic types of object with *fiat* object part aggregate – one object with *fiat* object part cluster and three out of 25 possible types of object with *fiat* object part group. ***Fiat*** **boundary** ***^mat^*** **:** demarcates *fiat* parts of a material entity; ***fiat*** **boundary** ***^immat^*** **:** demarcates *fiat* parts of an immaterial entity (i.e. a hole).

The distinction between groups and clusters requires to further differentiate the three basic types of aggregate of material entities discussed so far, i.e. object aggregate, *fiat* object part aggregate, and object with *fiat* object part aggregate, into respective types of clusters and groups (table 2; Fig. 6).

**Definition for ‘object group’:** *An* ***object group*** *is an object aggregate that is a mereological sum of spatially separated object entities, which do not adhere to one another through chemical bonds or physical junctions but, instead, relate to one another merely on grounds of metric proximity.*

**Definition for ‘object cluster’:** *An* ***object cluster*** *is an object aggregate that is a mereological sum of separate object entities, which adhere to one another through chemical bonds or physical junctions that go beyond gravity.*

**Explanation:** Whereas object groups are always demarcated by *fiat* boundaries*^immat^*, object clusters are demarcated exclusively by *bona fide* boundaries, but never by *fiat* boundaries*^immat^*. As a consequence, object aggregates can be demarcated by a mereological sum of either *bona fide* boundaries, if the aggregate is an object cluster (Fig. 5, left) or *bona fide* and *fiat* boundaries*^immat^*, if it is an object group (Fig. 4). In the latter case, the objects can be separated from one another through space or through other object entities that do not belong to the group.

At coarser levels of granularity, object clusters are demarcated by *bona fide* boundaries if they form objects at a coarser level of granularity, which implies that they are maximally self-connected and self-contained, possessing an internal unity and spatial discontinuity or qualitative heterogeneity towards their complement (e.g. the cell cluster comprising all cells of a multicellular organism is exclusively demarcated by *bona fide* boundaries). However, if they do not form objects at a coarser level of granularity (e.g., because the cell cluster does not include all cells of the multicellular organism), they are demarcated by *fiat* boundaries*^mat^* from other clusters of the same type (for definitions see table 2).

**Examples:** A shoal, a forest, a group of commuters on the subway, or the patients in a hospital are *object groups*, whereas the atoms constituting a molecule, the molecules forming the membrane of an animal cell, or the cells forming an epidermis in a human body are *object clusters*.

**Definition for ‘** ***fiat*** **object part group’:** *A* ***fiat object part group*** is a *fiat object part aggregate that is a mereological sum of spatially separated fiat object part entities, which do not adhere to one another through chemical bonds or physical junctions but, instead, relate to one another merely on grounds of metric proximity.*

**Definition for ‘** ***fiat*** **object part cluster’:** *A* ***fiat object part cluster*** is a *fiat object part aggregate that is a mereological sum of separate fiat object part entities, which adhere to one another through chemical bonds or physical junctions that go beyond gravity.*

**Examples:** The fingers of my left hand, opposing riverbeds, or the mainland of the Russian Federation are *fiat object part groups*, whereas a synapse, a hydrogen bond between two molecules, or the mainland of Turkey are *fiat object part clusters*.

**Definition for ‘object with** ***fiat*** **object part group’:** *An* ***object with fiat object part group*** *is an object with fiat object part aggregate that is a mereological sum of spatially separated object entities and fiat object part entities, which do not adhere to one another through chemical bonds or physical junctions but, instead, relate to one another merely on grounds of metric proximity.*

**Definition for ‘object with** ***fiat*** **object part cluster’:** *An* ***object with fiat object part cluster*** *is an object with fiat object part aggregate that is a mereological sum of separate object entities and fiat object part entities, all of which adhere to one another through chemical bonds or physical junctions that go beyond gravity.*

**Examples:** The equilibrium organ of lobsters, with the statolith (i.e. object) in the statocyst being surrounded by an arrangement of mechanoreceptors with their connected nerves (i.e. *fiat* object parts) that exhibit a specific spatial distribution is an *object with fiat object part group*. Other examples are a modern wireless cell phone connection or the territories of Turkey or of England. A human heart, a power outlet, a train station, or a traditional telephone cord connection between two telephones, on the other hand, are *object with fiat object part clusters*.

*Are groups and clusters of material entities already covered by BFO's ‘object’, ‘fiat object part’, or ‘object aggregate’?* Any type of aggregate of material entities, be it a group or a cluster, cannot be an object: groups of material entities are not objects, because their material entity parts are separated from each other by spatial gaps (Fig. 4). Clusters, like groups, are not objects, because they are not maximally self-connected and self-contained – while some object clusters can constitute *bona fide* objects at coarser levels of granularity, all their possible sub-clusters, each of which is an object cluster in its own right, constitute *fiat* object parts at coarser levels of granularity (Fig. 5).

Whereas object clusters and object groups can be subsumed under ‘object aggregate’, the differentiation of ‘object aggregate’ types that have the potential to constitute *bona fide* objects at coarser granularity levels from ‘object aggregate’ types that do not, would be lost. Moreover, groups and clusters of material entities other than objects are not covered by ‘object aggregate’ (see *Additional Top-Level Category: ‘Fiat Object Part Aggregate’* & *Additional Top-Level Category: ‘Object with Fiat Object Part Aggregate’*).

Whereas the distinction between clusters and *fiat* object parts is apparent, groups of material entities have a lot in common with *fiat* object parts. It has been noticed before that *fiat* boundaries come in two distinct types (those demarcating material entities and those demarcating immaterial entities) and, consequently, *fiat* entities may also be considered as: (i) “*fiat parts*” and (ii) “*fiat aggregates*”, the latter of which are aggregates of which the constituting material entities are not connected to each other [23]. This distinction has been discussed before, thereby referring to *fiat aggregates* as *fiat wholes*, *scattered (fiat) objects*, or as higher-order (*fiat*) objects [18]–[21]. These scattered entities correlate with our notion of ‘group’, and the “Hawaii-style” constitution of *“fiat wholes out of smaller bona fide parts”* ([20], p. 25) with our ‘object group’. The question to be answered at this point is, whether only *“– Montana-style – fiat parts within larger bona fide wholes”* are *fiat* object parts (i.e. a part of an object that is demarcated from this object by a *fiat* boundary*^mat^*; similar to the borderline of the state Montana, which does not follow any naturally given landmarks), or whether *“– Hawaii-style – fiat wholes out of smaller* bona fide *parts”* ([19], section 5.) are subsumed under ‘*fiat* object part’ as well. Following BFO's definition of ‘*fiat* object part’, however, the latter is not possible, since ‘*fiat* object part’ is defined as a material entity that is part of an object. Scattered material entities (i.e. groups), however, include gaps and immaterial entities, which are not necessarily part of an object.

#### Groups of Clusters and Groups of Groups

Due to the underlying differentiation between proximity and adherence, it is also possible to have aggregates that have both types of relations realized between their constituting parts. In other words, clusters of material entities can be spatially arranged in a certain proximity to one another forming **groups of clusters**, as for instance the distribution of simple pigment spot ocelli in a jellyfish or the distribution of polyplacophoran aesthetes (group of object with *fiat* object part cluster), each of which consists of several cells (i.e. objects) and its innervation (*fiat* object part), together forming an object with *fiat* object part cluster.

The necessity of a category ‘group of clusters’ is an immediate consequence of the ‘group’ category and the constitutive organization of material entities: whereas a colony of honeybees is an object group at the granularity level of multicellular organisms, it is a group of object clusters at the level of cells, because every single honeybee is a multicellular organism and at the same time a cell cluster. In the same way a heap of stones is an object group and also a group of molecule clusters, and a forest is a group of trees and also a group of cell clusters. As already mentioned above, whenever a specific spatial distribution of scattered material entities is important, the group category is required. In case these scattered entities are clusters, we need the category ‘group of clusters’. This is for instance the case with the visual sense system of a human being, which is a group of clusters consisting of a pair of spatially distributed eyes, each of which consists of a multiplicity of cells and the innervating nerves that together form an object with *fiat* object part cluster. Pairs of complex eyes of insects represent another example, together forming a group of two clusters of ommatidia and thus a group of object clusters. One is also dealing with a group of clusters if one evaluates the supply coverage of public transportation in a certain region and one has to consider the distribution pattern of train stations (i.e. group of object with *fiat* object part clusters) with their catchment areas. The distribution pattern of your synapses is also a group of *fiat* object part clusters.

Because metric proximity is relative (i.e. it refers to a spatial continuum) and can only be evaluated in relation to some external reference framework, it makes sense to talk about **groups of groups** in the same way as about groups of clusters. Different reference frameworks function like different granularity levels: for example, when considering the distribution pattern of different ciliary bands in a trochophora larvae (i.e. prototroch, neurotroch, telotroch), each band is a *fiat* object part group that together form a group of *fiat* object part groups. Another example is the distribution pattern of all honeybee colonies in a given region, with each colony being an object group and their distribution being a group of object groups. The same applies to the worldwide distribution of deciduous forests, which forms a group of object groups or, at a finer level of granularity, even a group of groups of cell clusters.

It follows that, whereas ‘object’ and ‘*fiat* object part’ represent the two primary building blocks for the first level of differentiation of ‘material entity’, the three possible basic types of cluster (i.e. ‘object cluster’, ‘*fiat* object part cluster’, ‘object with *fiat* object part cluster’; Fig. 6A, E, I) represent additional building blocks for groups of material entities, resulting in a total of five different building blocks for distinguishing different basic types of groups of material entity. All possible combinations of these five building blocks result in 31 (i.e. five different types of group-building blocks, each of which can be absent or present in the group, results in 5^2^ possible combinations minus the combination of the absence of all building blocks: 5^2^−1 = 31) basic types of groups of material entity (some of which are depicted in Fig. 6). If we also considered groups of groups, there would be even more.

## Discussion

### Suggestions for Extending the Basic Formal Ontology

**Figure 7:**
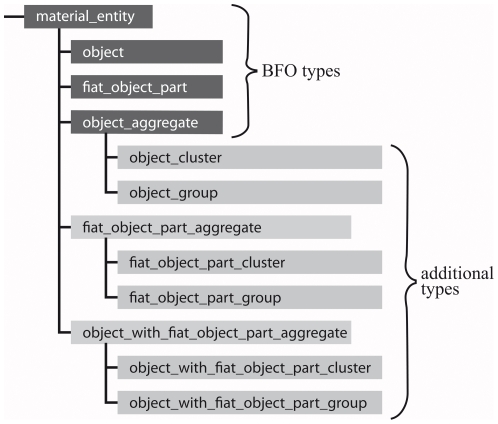
Taxonomy of top-level types of constitutively organized material entities. A taxonomy of top-level types of material entity and important subtypes that can be distinguished in constitutively organized material entities. types that are presently distinguished in the Basic Formal Ontology (BFO) are in dark grey.

The different types of material entity presented above are differentiated along three distinct lines of thought:

1. Given that the distinction between *bona fide* and *fiat* boundary is absolute and exhaustive for any given particular level of object granularity of constitutively organized material entities, it follows that:
  - ‘Object’ (i.e. material entity demarcated by a single continuous *bona fide* boundary) and ‘*fiat* object part’ (material entity demarcated by some *fiat* boundary) represent **primary building blocks** for all top-level types of material entity, and no other type of material entity has this role. As a consequence, a first level differentiation of basic types of material entity should exhaustively cover all possible combinations of these two primary building blocks. This results in five basic types of material entity (i.e. ‘object’, ‘*fiat* object part’, ‘object aggregate’, ‘*fiat* object part aggregate’, ‘object with *fiat* object part aggregate’; Fig. 3, 7). These five categories are disjunct: no material entity can instantiate more than one of these first level types at any given level of granularity.
  - By distinguishing material and immaterial entities, one can distinguish two types of *fiat* boundaries: ***fiat*** **boundary** ***^mat^*** that demarcates *fiat* parts of a material entity and ***fiat*** **boundary** ***^immat^*** that demarcates *fiat* parts of an immaterial entity (i.e. regions, cavities, tunnels, caves).
2. The distinction between *bona fide* and *fiat* boundary is only absolute for a given particular level of object granularity (see also *perspectivalism*, e.g. [11]), but is **granularity-dependent** across different levels of object granularity, because otherwise we would have to part with the assumption of a constitutive granularity of material entities. Since chemical bonds and physical junctions exist between *bona fide* objects, we can thus distinguish between:
  - **Topological coherence**: *fiat* object parts belonging to a particular object are continuously connected with one another and demarcated by *fiat* boundaries*^mat^*.
  - **Topological adherence**: objects belonging to a particular object cluster are in contact with one another through chemical bonds or physical junctions. Whereas each object belonging to the cluster is demarcated by a single *bona fide* boundary, the cluster as a whole is demarcated by a mereological sum of the *bona fide* boundaries of its object entity parts. At a coarser level of granularity, topological adherence is treated as topological coherence and sub-clusters of an object cluster as *fiat* object parts of the corresponding object of coarser granularity (Fig. 5), which in their turn are demarcated from one another by *fiat* boundaries*^mat^*.
  - **Metric proximity**: objects belonging to a particular object group are separated from each other either by space (even if it is infinitesimally small) or by other objects – no relevant adherence-forces other than gravity are in effect between the object entity parts of the group. Whereas each object belonging to the group is demarcated by a single *bona fide* boundary, the group as a whole is demarcated by a combination of *bona fide* boundaries of its object entity parts and *fiat* boundaries*^immat^* across the space separating the object entity parts from each other (Fig. 4, 6).
  As a consequence, we can distinguish two subtypes for each basic type of material entity aggregate: **groups** of material entities and **clusters** of material entities (Fig. 6). The relation between material entities of a group is characterized by metric proximity, whereas in clusters it is characterized by topological adherence. Moreover, contrary to clusters, groups are always demarcated by some *fiat* boundary*^immat^*.
3. When considering possible types of configurations and patterns of spatial distribution of material entities, we can distinguish five **material building blocks** for differentiating 31 different types of *groups* of material entities, i.e. the two primary material building blocks, ‘object’, ‘*fiat* object part’, and three additional material building blocks, ‘object cluster’, ‘*fiat* object part cluster’, ‘object with *fiat* object part cluster’.

Obviously, only one of the types of possible combinations of primary building blocks is also covered by BFO: ‘object aggregate’, which is a type of material entity that results from the aggregation of several *bona fide* objects (Fig. 3C). The other two possible combinations (i.e. ‘*fiat* object part aggregate’, Fig. 3D; ‘object with *fiat* object part aggregate’, Fig. 3E) are not covered by BFO. Moreover, BFO covers none of the types differentiating material entity aggregates into groups and clusters. However, we have provided examples that demonstrate that these additional types of material entity actually exist, and we have argued that they are important in various scientific domains.

Although, in general, the different categories of BFO are defined so as to be mutually exclusive relative to a given level of granularity, it also has been explicitly stated that the types of material entity BFO distinguishes do not exhaustively cover all theoretically possible instances of material entity [11]. However, the types of material entity not covered by BFO were considered to *“lack salience and are systematically irrelevant for a principled analysis of the ontology of scientific domains”*, which is the reason why *“they are intentionally not included in any existing definition sets, taxonomies or implementations of BFO”* ([11], p. 71). Experience has shown that this assessment is wrong (e.g. the example of ‘synapse’ being subsumed under ‘*fiat* object part’ and ‘object aggregate’, because BFO lacks the category ‘*fiat* object part aggregate’; [15]) and we provided several examples across different scientific domains for various types of material entity not covered by BFO. Therefore, we suggest to extend BFO category of ‘material entity’ to include, besides the subcategories ‘object’, ‘*fiat* object part’, and ‘object aggregate’, also ‘*fiat* object part aggregate’ and ‘object with *fiat* object part aggregate’ (Fig. 7). Moreover, we suggest to further include ‘object cluster’ and ‘object group’ as the subcategories of ‘object aggregate’, ‘*fiat* object part cluster’ and ‘*fiat* object part group’ as the subcategories of ‘*fiat* object part aggregate’, and ‘object with *fiat* object part cluster’ and ‘object with *fiat* object part group’ as the subcategories of ‘object with *fiat* object part aggregate’ (Fig. 7).

The need for extending BFO becomes also apparent when taking a look at the anatomy ontologies currently listed as OBO Foundry candidate ontologies (c.f. http://www.obofoundry.org/): many of them diverge from BFO's top-level organization of material entity sub-categories. Often, categories like ‘anatomical cluster’ and ‘anatomical system’ are introduced as basic categories (see e.g. Common Anatomy Reference Ontology, Version 1.5; Drosophila gross anatomy, Version 1.40; Teleost Anatomy Ontology, Version 1.205), which are comparable to the here proposed distinction between cluster and group. Other anatomy ontologies make no top-level distinction between subcategories of material entity at all and, instead, list a multiplicity of anatomical structure categories at the same basic taxonomic level, irrespective of their degree of generality. The Uber anatomy ontology (Version 1.93), for example, lists more than 150 basic categories of ‘anatomical structure’, including general categories like for instance ‘cell part’ alongside with much more specific categories such as ‘Bachmann’s Bundle’ or ‘retina photoreceptor layer inner segment’. This organization might have been chosen by the developers of this ontology due to the lack of BFO providing the adequate basic categories for material entity. The use of the here proposed additional top-level categories of material entity could significantly clear up this unorganized set of general and more specific categories.

Extending BFO with these additional categories is not problematic insofar as the new categories will be added as new leave categories to the ontology (Fig. 7). Consequently, all existing ontologies that have been developed with BFO as top-level template are compatible with the here proposed extended BFO. Those ontologies that benefit from the additional categories, however, will have to make the appropriate changes using respective tools and techniques [34]–[39] in order to take advantage of the extended BFO.

Since the here suggested extension of BFO only introduces one additional taxonomic level within the class-subclass hierarchy of categories of material entity (Fig. 7), this extension will not make the respective ontologies more difficult to reason with algorithmically, but could significantly improve their usability in annotations. Above all, however, it will improve the overall compatibility of respective biomedical ontologies, because the problematic categories (e.g. synapse), which so far have been arbitrarily subsumed, could be subsumed under the same basic categories across all these ontologies.

### Conclusions

We have shown that, under the premise of a constitutive granularity of material entities, the current top-level categories of material entity of BFO are insufficient for developing domain ontologies that are consistent with the single inheritance policy. Only by adding two further top-level categories of material entity to BFO, they become mutually exclusive relative to a given level of granularity. With the suggested extension, formerly problematic classes such as ‘synapse’ can be subsumed unambiguously under one of the additional categories (‘*fiat* cell part aggregate’ in case of ‘synapse’).

However, while the proposed extensions to BFO do justice to constitutively organized material entities, most biological material entities exhibit what is generally referred to as cumulative constitutive organization (e.g. [26], [28], [40], [41], see also *somatic hierarchy* sensu [42]). Contrary to a constitutive granularity, in a cumulative constitutive granularity *not all* the objects belonging to one level of granularity form parts of objects of the next higher level of granularity: not all atoms are parts of molecules (e.g. ions, chlorine radicals), not all molecules are parts of cells (e.g. extracellular matrix, a macromolecular formation that is a component of tissues and organs that is located outside of cells), and not all cells are parts of organs (e.g. erythrocytes, coelomocytes, leukocytes). Thus, it remains to be evaluated and assessed, whether this extended top-level categorization of material entity is also exhaustive and mutually disjoint for cumulative-constitutively organized material entities.
